# Oncology pharmacists’ response to COVID-19 pandemic in Jordan: The King Hussein Cancer Center experience

**DOI:** 10.7189/jogh.11.03048

**Published:** 2021-03-27

**Authors:** Abeer A Al-Rabayah, Suzan S Hammoudeh, Rasha S AbuBlan, Rula Najjar, Sewar S Salmany, Noor Nassar, Lama Nazer, Saad M Jaddoua

**Affiliations:** Department of Pharmacy, King Hussein Cancer Center. Amman, Jordan

Coronavirus disease (COVID-19) is a new communicable disease that is caused by the novel Severe Acute Respiratory Syndrome Coronavirus SARS-CoV-2. Wuhan, the capital of Hubei, China, reported the first human infection case in December 2019 [1]. On 12 March 2020, the World Health Organization declared COVID-19 disease as a pandemic and an international public health emergency [2]. Jordan reported its first COVID-19 case on the 2 March 2020 [3]. The Jordanian government took essential public health measures to contain the spread of the virus in Jordan. The ministry of health has launched an official website for the COVID-19 pandemic targeting the public and providing general guidance documents to hospitals, workplaces, companies, families, and travelers. However, there was no specific guidance for oncology hospital pharmacies regarding response requirements to the COVID-19 pandemic [4].

Hospital pharmacists are among the frontline health care workers who are participating in the fight against this virus. Therefore, they must be fully prepared to maintain the continuity of care under such an emergency outbreak [5]. Despite having international guidance documents for hospital pharmacists, the World Health Organization, recommend that response plans are context-specific and should be developed to match countries’ population mix and public health needs [6-9].

This paper aims to share the COVID-19 pandemic response model of the pharmacy department at King Hussein Cancer Center (KHCC) in Jordan along with lessons learned, encountered challenges and future directions.

## RESPONSE 1: MAINTAINING PATIENT CARE

At KHCC, an electronic medical record system is already in use since 2014. Having this system facilitated the implementation of virtual prescription processing by some pharmacists from home to limit the number of pharmacists on duty while maintaining the continuity of pharmaceutical care. Furthermore, patient counseling and medication reconciliations have been provided through telepharmacy to maintain the safe and rational use of medicines by our patients, and to minimize patient-pharmacist direct contact time.

The outpatient service has been implementing the medications’ home delivery service system since 2018. Therefore, this helped in the rapid expansion of the service to reach all patients. This service protected patients from unnecessary travel to the center and minimized the patients’ flow into the center.

Narcotics and controlled medications are high-risk items that are regulated by national regulatory agencies. At KHCC, despite having the home delivery service for medications, narcotic and controlled substances cannot be delivered to patients’ homes according to the Jordan Food and Drug administration law for narcotics and controlled medications [10]. To tackle this challenge, the pharmacy department created a drive-through dispensing window to dispense narcotics and controlled medications.

## RESPONSE 2: MAINTAINING THE SAFETY OF PHARMACY STAFF

KHCC pharmacy staff from all sections were divided into teams A and B to facilitate providing service and minimize cross interaction. Each team was assigned specific days to work from the hospital without any interaction or meetings between the two groups. If a team member is suspected of having COVID-19, then the whole group can be quarantined and the other group takes over. The pharmacy administration organized the staffing of teams in a way that allows longer hours per day with more days off during the week to minimize days of contact. In addition to on-ground frontline teams, a work-from-home team provided backup support to their frontline colleagues.

The infection control program in KHCC, instructed pharmacy staff on safety measures (1-2 m social distance, hand hygiene, surface disinfection, using masks and gloves) to protect themselves and their families; those who had been traveling or were in contact with someone who had been traveling were given two-week home isolation leave

## RESPONSE 3: MAINTAINING MEDICATION SUPPLY

Medication shortages are a growing risk that might impact patients’ access to these essential items. Supply chain management guidelines are needed to respond to drug shortages, especially when it comes to oncology medications, where alternatives might be limited [11-13].

KHCC procurement section works with suppliers using the Kraljic matrix categories and clinical inputs from health care providers to manage potential supply risks. The Kraljic matrix is a strategic tool that was developed by Peter Kraljic to guide managers to formulate strategies for guarding against supplies disruption and maintaining medications supply. It facilitates making strategic decisions to maintain the supply of medications. It categorizes suppliers into routine, strategic, leverage, or bottleneck based on cost and risk impacts [14,15]. For example, bottleneck items are low-cost items, but any shortage is associated with high risk, on the other hand strategic items are of high cost and high risk. Maintaining safety stock levels of critical items (strategic and bottleneck) is routinely conducted at KHCC to recognize any medication shortage risk at an early stage. Besides, maintaining sustainable professional partnerships with suppliers is a key strategic goal to mitigate drug shortage risks at an early stage too.

**Figure:**
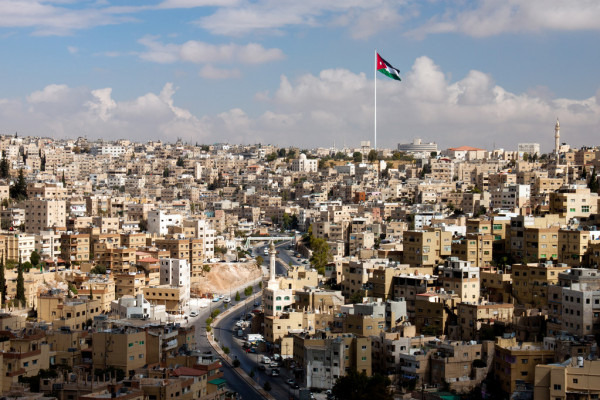
Photo: © Abdulla Ghatasheh, retrieved from: https://www.pexels.com/search/jordan.

Under the COVID-19 pandemic, medication supply challenges were considered a significant threat to patients’ access to medicine. Therefore, the procurement section at the pharmacy department took strategic preventive measures building on the drug shortages management SOPs mentioned earlier to mitigate any medication shortage risks that might happen during the pandemic. Prioritizing clinical needs and suggesting alternatives was the first primary step. All pharmacy sections worked together to modify and update the lifesaving and critical medication lists to form an emergency formulary list. For an oncology center, the Bone Marrow Transplant List, the ICU medications list, the anti-infective list, and the essential antineoplastic list that includes curative regimens formed the emergency formulary.

Six to twelve-month supply was ordered from the emergency formulary as it included medications with limited alternatives, and many of them required direct importation. It is essential to follow up on international alerts to identify any emerging risk at an early stage. At KHCC, we apply the just in time or more lean stock control methods in supply management. However, under the COVID-19 public health crisis, there was a need to maintain higher stocks of some medications from the emergency formulary list to match that exceptional situation.Forecasting becomes complicated when we identify risks, with both demand and supply, becoming harder to predict. Therefore, close monitoring must be done. The weekly stock of the emergency formulary medications was reviewed by the institutions’ supply committee to make sure that supply is maintained and to take corrective actions when needed promptly.

## RESPONSE 4: MULTI-STAKEHOLDER COLLABORATION

KHCC pharmacy is continuously working with partners such as the JFDA, customs, the pharmaceutical industry, and other hospitals to maintain medication supply in Jordan. Building on such collaborations is essential, especially under public health emergencies, to maintain access to medicines.

## RESPONSE 5: EDUCATION

Education sessions about COVID-19 were delivered to all pharmacy staff by the infection control team at KHCC to educate them about the virus, methods of transmission, and how pharmacists can protect themselves. A virtual training about critical care for non-ICU clinicians was also shared with all pharmacy staff to stay updated regarding COVID-19 management.

## RESPONSE 6: RAPID ACCESS TO DRUG INFORMATION

The pharmacy department, in collaboration with KHCC medical library, provides all health care providers with a point of care access to online formulary and drug information resources.Having rapid access to drug information helped pharmacists to actively participate in sharing COVID-19 drug information updates with other health care providers.

## REFLECTIONS, LESSONS LEARNED AND CHALLENGES

We have learned from this experience that teamwork, partnership, national and international collaboration are core precursors for rapid actions. Also, applying telepharmacy and more virtual processes might be an opportunity for improving the delivery of pharmacy services in the future for oncology and general hospital pharmacy.

On the other hand, the pharmacy department has encountered some challenges; the availability of staff transportation, especially that the country was under lockdown, and there was no public transportation. However, the KHCC administration addressed this challenge by providing accommodation for staff in nearby hotels.

As nurseries and schools were closed, the pharmacy schedule was adjusted to provide working parents who both need to be on duty with an opportunity to organize their working time in a way that does not affect their children.

## FUTURE DIRECTION FOR HOSPITAL PHARMACY

Engaging hospital pharmacists in providing public health services in Jordan and the region in the future is essential. Pharmacy associations and hospitals should provide continuous education for pharmacists to implement this role. Recently, the US FDA has authorized pharmacists to conduct COVID-19 testing, and this means that pharmacists can have expanded roles during disease outbreaks and global pandemics [16]. In the future, hospital pharmacists, including oncology pharmacists, may be involved in providing population-based care, health education, and having an active role in public health policy [17].

Moreover, as the antimicrobial resistance is increasing and communicable diseases will not vanish, more infectious diseases specialized pharmacists are needed. Residency programs might also add infectious disease and pharmacy public health rotations to their programs as core rotations. Also, access to IT solution should be optimized to enable more hospital pharmacists to provide patient care under any situation.

In addition, having a department specific wellness and resilience program is essential to provide staff with all required psychological support. Therefore, the pharmacy department released a pharmacy wellness policy during 2020.

In conclusion, maintaining patient care, maintaining pharmacy staff safety, maintaining medication supply, multi-stakeholder collaboration, education, and rapid access to drug information are considered the minimum requirements for pandemic response plans for oncology hospital pharmacies. However, we believe that non-oncology hospital pharmacies can also adapt our model to their settings and use it to respond to pandemics.

## References

[1] Ahn D-G Shin H-J Kim M-H Lee S Kim H-S Myoung J Current Status of Epidemiology, Diagnosis, Therapeutics, and Vaccines for Novel Coronavirus Disease 2019 (COVID-19). J Microbiol Biotechnol. 2020;30:313-24. 10.4014/jmb.2003.03011 32238757 PMC9728410

[2] World Health Organization. WHO Director-General’s opening remarks at the media briefing on COVID-19 - the 11th of March 2020. Available: https://www.who.int/emergencies/diseases/novel-coronavirus-2019?gclid=EAIaIQobChMIn_LchNii7wIVze3mCh0G8gYlEAAYASAAEgKlaPD_BwE. Accessed: 14 April 2020.

[3] World Health Organization. Eastern Mediterranean Region Covid-19 affected countries.2020. Available: https://app.powerbi.com/view?r=eyJrIjoiN2ExNWI3ZGQtZDk3My00YzE2LWFjYmQtNGMwZjk0OWQ1MjFhIiwidCI6ImY2MTBjMGI3LWJkMjQtNGIzOS04MTBiLTNkYzI4MGFmYjU5MCIsImMiOjh9.Accessed: 14 April 2020.

[4] Ministry of HealthAvailable: https://corona.moh.gov.jo/ar. Accessed: 30 July 2020.

[5] Liu S Luo P Tang M Hu Q Polidoro JP Sun S Providing pharmacy services during the coronavirus pandemic. Int J Clin Pharm. 2020;42:299-304. 10.1007/s11096-020-01017-0 32222911 PMC7101875

[6] American Society of Hospital Pharmacists ASHP. Pharmacy Readiness for Coronavirus Disease 2019 (COVID-19) Recommendations for state policy makers. 2020. Available: https://www.ashp.org/-/media/assets/advocacy-issues/docs/Pharmacy-Readiness-for-Coronavirus-Disease-2019-COVID-19-STATE. Accessed: 14 April 2020.

[7] International Pharmaceutical Federation (FIP). COVID-19 pandemic: Guidelines for pharmacists and the pharmacy workforce. 2020. Available: https://www.fip.org/files/content/priority-areas/coronavirus/COVID-19-Guidelines-for-pharmacists-and-the-pharmacy-workforce.pdf. Accessed: 14 April 2020.

[8] World Health Organization. 2019 Novel Coronavirus (2019-n CoV): Strategic Preparedness and Response Plan. 2020. Available: https://www.who.int/docs/default-source/coronaviruse/srp-04022020.pdf. Accessed: 14 April 2020.

[9] World Health Organization. Coronavirus disease 2019 (COVID-19) strategic preparedness and response plan: accelerating readiness in the Eastern Mediterranean Region. Cairo: WHO Regional Office for the Eastern Mediterranean; 2020.Available: Available: https://applications.emro.who.int/docs/EMCSR260E.pdf?ua=1. Accessed: 14 April 2020.

[10] Jordan Food and Drug Administration. Narcotics and Controlled Substances drug law. Available: https://applications.emro.who.int/docs/EMCSR260E.pdf?ua=1. Accessed: 14 April 2020.

[11] Valgus J Singer EA Berry SR Rathmell WK Ethical challenges: managing oncology drug shortages. J Oncol Pract. 2013;9:e21. 10.1200/JOP.2012.000779 23814521 PMC3595447

[12] American Society of Hospital Pharmacists ASHP. Guidelines on Managing Drug Product Shortages. Available: https://www.ashp.org/-/media/assets/policy-guidelines/docs/guidelines/managing-drug-product-shortages.ashx. Accessed: 14 April 2020.

[13] Lysons K, Farrington B. Purchasing, and Supply Chain Management. London: Pearson Education; 2006.

[14] Caniëls MCJ Gelderman CJ Purchasing strategies in the Kraljic matrix—A power and dependence perspective. J Purchasing Supply Manage. 2005;11:141-55. 10.1016/j.pursup.2005.10.004

[15] The Chartered Institute of Procurement & Supply (CIPS). How to Appraise Suppliers. (2007). Available: https://www.cips.org/Documents/Resources/Knowledge%20How%20To/How%20to%20Appraise%20Suppliers.pdf. Accessed: 14 April 2020.

[16] U.S. Department of Health & Human Services Office of the Assistant Secretary for Health. Guidance for Licensed Pharmacists, COVID-19 Testing, and Immunity under the PREP Act. 2020. Available: https://www.hhs.gov/sites/default/files/authorizing-licensed-pharmacists-to-order-and-administer-covid-19-tests.pdf. Accessed: 14 April 2020.

[17] American Society of Health-System Pharmacists ASHP Statement on the Role of Health-System Pharmacists in Public Health. Am J Health Syst Pharm. 2008;65:462-7. 10.2146/ajhp070399 18281740

